# Supplementary material for: Adsorptive Removal of Azithromycin Antibiotic from Aqueous Solution by Azolla Filiculoides-Based Activated Porous Carbon
Source: Nanomaterials (Basel). 2021 Dec 3;11(12):3281. doi: 10.3390/nano11123281 (PMC8709189; doi:10.3390/nano11123281)
Supplement: Supplementary file 1 [file nanomaterials-11-03281-s001.zip › nanomaterials-1415114-Supplementary Materials (New).pdf]

nanomaterials

MDPI

Supporting information

Adsorptive Removal of Azithromycin Antibiotic from Aqueous Solution by Azolla Filiculoides-based Activated Porous Carbon

Davoud Balarak [1,*], Amir Hossein Mahvi [2], Saeideh Shahbaksh [3], Md A. Wahab [4,*] and Ahmed Abdala [5,*]

1 Department of Environmental Health, Health Promotion Research Center, Zahedan University of Medical Sciences, Zahedan 9816743463, Iran
2 Center for Solid Waste Research, Institute for Environmental Research, Tehran University of Medical Sciences, Tehran 1417653911, Iran; ahmahvii@gmail.com
3 Student Research Committee, Zahedan University of Medical Sciences, Zahedan 9816743463, Iran; dbchemistry2@gmail.com
4 Institute for Advanced Study, Chengdu University, Sichuan, Chengdu 610106, China
5 Chemical Engineering Program, Texas A&M University at Qatar, Doha POB 23874, Qatar
* Correspondence: dbalarak2@gmail.com (D.B.); mawahab@gmail.com (M.A.W.); ahmed.abdala@qatar.tamu.edu (A.A.)

The Langmuir adsorption model is based on monolayer adsorption that exclusively occurs at defined fixed-in number, equivalent, and identical sites. The Langmuir isotherm is expressed by equation 1 [1]:

$$\frac{C_e}{q_e} = \frac{1}{q_m K_L} + \frac{C_e}{q_m} \tag{1}$$

where q_m (mg/g) is the maximum amount of AZM adsorbed per unit weight of the AFAC; q_e (mg/g) is the equilibrium adsorption capacity, and K_L (L/mg) is the Langmuir constant that is related to the affinity of the binding sites. q_m represents the practical limit of the adsorption capacity when the surface is fully covered with AZM molecules. Hence, it is used to compare the performance of different adsorbents. q_m and K_L are calculated from the intercept and slope of a straight-line fitting of C_e/q_e versus C_e (Figure S1). Moreover, R_L is a dimensionless separation factor or equilibrium parameter given by the following equation [2]:

$$R_L = \frac{1}{1+C_0 K_L} \tag{2}$$

This parameter indicates the isotherm is favorable (R_L<1), unfavorable (R_L>1), irreversible (R_L= 0), or linear (R_L= 1).

Figure S1 shows that the Langmuir isotherm cannot describe the adsorption process as Ce/qe vs. Ce results at different adsorption temperatures do not follow a linear trend.

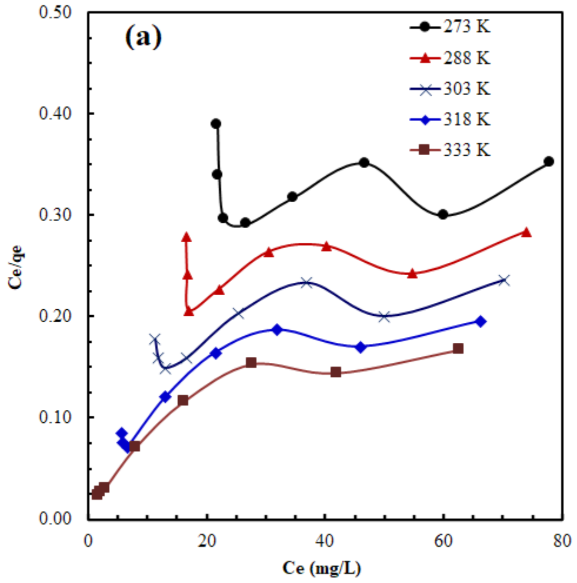

Figure S1. The plot C_e/q_e versus C_e based on Langmuir isotherm.

Temkin model assumes a uniform distribution of surface binding energy, and that for all the molecules, the adsorption heat decreases linearly upon an increase in the adsorbent surface coverage; this model is expressed as follows [3]:

$$q_e = \beta \ln \alpha + \beta \ln C_e \qquad \beta = \frac{RT}{b} \tag{3}$$

where α is the binding constant of equilibrium (L/g) identical to the extreme binding energy, b is Temkin isotherm constant and is related to the adsorption heat (J/mol), R is the universal gas constant (8.314 J/mol K), and T is the absolute temperature (K). α and b are the isotherm constants that can be evaluated from q_e versus $\ln C_e$'s plot.

For Temkin isotherm, α values of 13.28 to 1.124 L/mg at different temperatures show that AZM has higher adsorption potential or binding potential to the AFAC. The Temkin heat of adsorption β values of 72.9 to 39.55 J/mol at different temperatures and less than 80 KJ/mol suggests favorable physical adsorption [4].

D-R isotherm data was connected so as is to recognize physical and chemical adsorption through adsorption energies. D-R model is expressed as equation 4 [5]:

$$\ln q_e = \ln q_m - \beta \varepsilon^2 \tag{4}$$

where β (mol^2/kJ^2) is the activity coefficient associated with the average free adsorption energy per mole of AZM when transferred from infinity to the surface of solids in solution, q_m (mg/g) is a theoretical monolayer and capacity saturation, and ε (J/mol) is the Polanyi potential determined by equation 5 [6]:

$$\varepsilon = RT \ln \left(1 + \frac{1}{C_e}\right) \tag{5}$$

The average free adsorption energy E (kJ/mol) is calculated using equation 6 [7]:

$$E = (-2K)^{-\frac{1}{2}} \tag{6}$$

From D-R isotherm, the value of E is used to estimate the type of adsorption mechanism. If the E value is between 8 and 16 kJ/mol, this indicates chemical adsorption, while the E value is <8 kJ/mol; the adsorption process is physical adsorption [8]. E's mean squared energy values for this study are between 0.07 to 0.845 kJ/mol, again suggesting the successful physical adsorption.

The difference in q_m obtained by Langmuir and D-R model may be due to differences in the definition of maximum capacity in the two models. In the Langmuir model, q_m represents the maximum adsorption of AZM in the monolayer layer. Still, in the D-R model, q_m represents the maximum adsorption of AZM molecules' total specific micropore volume of the AFAC [9]. Therefore, the obtained results indicate the adsorption occurs preferably on the micropores rather than on the AFAC surface.

Kinetics

The kinetic of the AZM adsorption on AFAC is analyzed using the pseudo-first-order kinetic model, the pseudo-second-order model, and the intra-diffusion model. The integrated form of the pseudo-first-order kinetic equation is given by [10]:

$$ln(q_e - q_t) = ln\, q_e - K_1 t \quad (7)$$

Where q_e is the equilibrium sorption uptake, q_t (mg/g) is the amount of adsorbed AZM on the AFAC at time t, and K_1 (1/min) is the rate constant of the first-order adsorption. q_e is extrapolated from the experimental data at time t =∞. A straight line of Ln (q_e-q_t) versus t suggests the applicability of this kinetic model (Figure S2). K_1 and q_e can be determined from the slope and intercept of the plot, respectively.

The pseudo-second-order kinetic rate expression in the integrated form is [11]:

$$\frac{t}{q_t} = \frac{1}{k_2\, qe^2} + \frac{t}{qe} \quad (8)$$

Where K_2 is second-order rate constants (g/mg.min). The values of different parameters determined from pseudo-second-order and pseudo-first-order kinetic models for AZM ions with their corresponding correlation coefficients are presented in Table 1S and (Figure S3). The experiments were performed at five different temperatures and with the initial AZM concentration of 100 mg/L. The correlation coefficients of the second-order kinetic model are very close to 1, showing that this model is better in explaining the adsorption kinetics of the present study.

Pseudo-first-order kinetic and pseudo-second-order kinetic models cannot determine the diffusion mechanism. About the diffusion mechanism using the adsorption kinetics data. The model is expressed as The intra-particle diffusion as a mechanistic model was used to give some information follows [12]:

$$q_t = K_d\, t^{\frac{1}{2}} + C \quad (9)$$

Where C is the intraparticle diffusion constant, K_d (mg/g.min$^{0.5}$) is the rate constant for intraparticle diffusion. If a straight line resulted from plotting q_t versus $t^{0.5}$, then the adsorption's overall process is exclusively limited to intraparticle mass transfer.

However, if more than one linear plot resulted from plotting the data, then more than one stage of the previously mentioned stages strongly affects adsorption's overall process. The existence of a multiline in the plot (Figure S4) indicates several mechanisms that control the adsorption process. The first step, which has a steeper slope, is named the external surface adsorption or momentary adsorption step. The gradual absorption occurs in the second step when the intra-particle diffusion phenomenon controls the rate of adsorption. Finally, intra-particle diffusion is reduced in the third step due to the very slow adsorption rate [13]. Figures S2–4 show the **a**dsorption kinetics for the adsorption of AZM on AFAC at under various conditions.

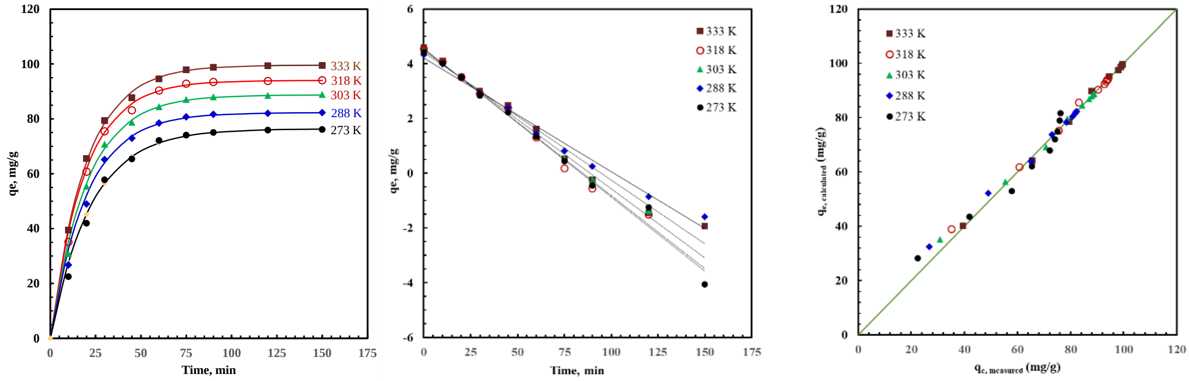

Figure S2. The Adsorption kinetics for the adsorption of AZM on AFAC at various temperatures. a) qe vs. time, the Symbols represent the experimental results, and the curves represent the pseudo-first-order model fitting, b) the linearized (integral form) of the pseudo-first-order model, and c) the model calculated adsorption capacity vs. the experimentally measured adsorption capacity shown the excellent agreement as the data points are on or very near to the 45° line.

From pseudo-second-order rate constants (K_2) and using the Arrhenius equation (Equation 10), it is possible to gain some insight into the type of adsorption [14].

$$\ln k_1 = \ln A - \frac{E}{RT} \qquad (10)$$

where E is the activation energy (J/mol), A the temperature-independent Arrhenius factor (g/mol.s). The slope of the plot of *ln* K_2 vs. 1/T can then be used to evaluate E. Low activation energies (0–40 KJ/mol) are characteristic of physical adsorption, while higher ones (40–800 kJ/mol) suggest chemisorption. The present results give E= 0.033 KJ/mol for the adsorption of AZM onto AFAC, indicating that the adsorption process has a low potential barrier and therefore corresponds to physisorption.

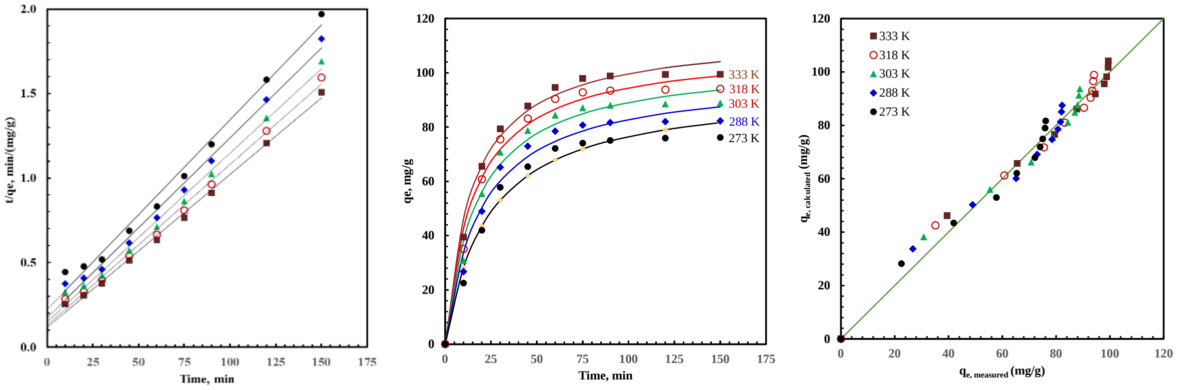

Figure S3. The Adsorption kinetics for the adosprion of AZM on AFAC at various temperatures. The Symbols represent the experimental results, and the curves represent the pseudo-second-order model fitting. The inset shows the linear form of the pseudo-second-order model.

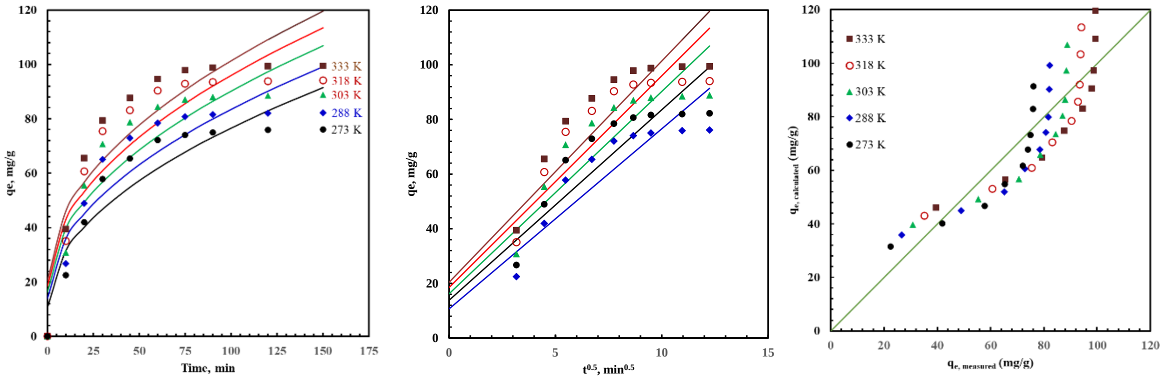

Figure S4. The Adsorption kinetics for the adsorption of AZM on AFAC at various temperatures. The Symbols represent the experimental results, and the curves represent the inter-particle diffusion model fitting. The inset shows the linear form of the inter-particle diffusion model.

Using the Arrhenius equation (Equation 11), the pseudo-first-order rate constant (k_1) at different adsorption temperatures, the frequency factor (A), and the activation energy (E) are determined.

$$\ln k_1 = \ln A - \frac{E}{RT} \quad (11)$$

and it is possible to gain some insight into the type of adsorption [14]

Table S1: Kinetic parameters for the adsorption of AZM on AFAC

T (K)	Pseudo-first-order			Pseudo-second-order			Inter-particle diffusion		
	k_1 min^{-1}	qe mg/g	R^2	k_2 $(mg/g)^{-1}min^{-1}$	Qe, mg/g	R^2	k_d $(mg/g)min^{-1/2}$	C mg/g	R^2
273	0.0449	76.3	0.995	*4.51x10^-4^* 4.51×10^{-4}	94.46	0.983	6.59	0.15	0.863
288	0.0502	82.3	0.990	5.26 $x10^{-4}$	98.72	0.987	6.97	0.14	0.843
303	0.0505	88.7	0.996	5.52 $x10^{-4}$	104.49	0.991	7.41	0.14	0.840
318	0.0534	94.0	0.989	5.85 $x10^{-4}$	109.20	0.993	7.75	0.13	0.834
333	0.0517	99.6	0.995	5.91 $x10^{-4}$	114.37	0.996	8.10	0.12	0.836

1. Ding, R.; Zhang, P.; Seredych, M.; Bandosz, T.J. Removal of antibiotics from water using sewage sludge- and waste oil sludge-derived adsorbents. *Water Res.* **2012**, *46*, 4081–4090.
2. Bui, T.X.; Choi H. Adsorptive removal of selected pharmaceuticals by mesoporous silica SBA-15. *J. of Hazard. Mater.* **2009**; *168*, 602–608.
3. Moussavi, G.; Alahabadi, A.; Yaghmaeian, K.; Eskandari, M. Preparation, characterization and adsorption potential of the NH4Cl-induced activated carbon for the removal of amoxicillin antibiotic from water. *Chem. Eng. J.* **2013**, *217*, 119–128.
4. Guler, U.A.; Sarioglu, M. Removal of tetracycline from wastewater using pumice stone: equilibrium, kinetic and thermodynamic studies. *J. of Environ. Health Sci. & Eng.* **2014**, *12*, 79–87.
5. Kerkez-Kuyumcu, Ö.; Bayazit, Ş.S.; Salam, M.A. Antibiotic amoxicillin removal from aqueous solution using magnetically modified graphene nanoplatelets. *J. of Ind. and Eng. Chem.* **2016**, *36*, 198–205.
6. Kim, S.H.; Shon, H.K.; Ngo, H.H. Adsorption characteristics of antibiotics trimethoprim on powdered and granular activated carbon. *J. of Ind. and Eng. Chem.* **2010**, *16*, 344–349.

7. Kyzas, G.Z.; Bikiaris, D.N.; Seredych, M.; Bandosz, T.J.; Deliyanni, E.A. Removal of dorzolamide from biomedical wastewaters with adsorption onto graphite oxide/poly(acrylic acid) grafted chitosan nanocomposite. *Bioresour. Technol.* **2014**, *152*, 399–406, doi:10.1016/j.biortech.2013.11.046.
8. Azarpira, H.; Mahdavi, Y.; Khaleghi, O. Thermodynamic Studies on the Removal of Metronidazole Antibiotic by Multi-Walled Carbon Nanotubes. *Der Pharm. Lett.* **2016**, *8*, 107–113.
9. Yu, F.; Li, Y.; Han, S.; Ma, J. Adsorptive removal of antibiotics from aqueous solution using carbon Materials. *Chemosphere* **2016**; *153*, 365–385.
10. Liu, Z.; Xie, H.; Zhang, J.; Zhang, C. Sorption removal of cephalexin by HNO3 and H2O2 oxidized activated carbons. *Sci. China Chem.* **2012**; *55*, 1959–1967.
11. Erşan, M.; Bağd, E. Investigation of kinetic and thermodynamic characteristics of removal of tetracycline with sponge like, tannin based cryogels. *Colloids and Surf. B: Biointerfaces* 2013, 104, 75–82.
12. Gao, Y.; Li, Y.; Zhang, L.; Huang, H.; Hu, J.; Shah, S.M.; Su, X. Adsorption and removal of tetracycline antibiotics from aqueous solution by graphene oxide, *J. Colloid. Interface Sci.* **2012**, *368*, 540–546.
13. Liu, W.; Zhang, J.; Zhang, C.; Ren, L. Sorption of norfloxacin by lotus stalk-based activated carbon and iron-doped activated alumina: mechanisms, isotherms and kinetics. *Chem. Eng. J.* **2011**; *171*, 431–438.
14. Hu, D.; Wang, L. Adsorption of amoxicillin onto quaternized cellulose from flax noil: Kinetic, equilibrium and thermodynamic study. *J. of the Taiwan Inst. of Chem. Eng.* **2016**; *64*, 227–234.
